# Best Practices for Clinical and Translational Research and Implementation

**DOI:** 10.1111/cts.12411

**Published:** 2016-09-12

**Authors:** DL Kroetz

**Affiliations:** ^1^ Department of Bioengineering and Therapeutic SciencesUniversity of California San FranciscoSan FranciscoCaliforniaUSA

Scientists in all sectors have been raising concerns in recent years about the lack of reproducibility of biomedical research results. An analysis of in‐house validation efforts at Bayer HealthCare found that <25% of potential targets could be reproduced in‐house.^1^ Similarly, of 53 “landmark” studies selected because they described completely new findings, only 11% could be confirmed in‐house by Amgen scientists.^2^ Such dismal numbers have raised concern among scientists in all sectors about the lack of transparency in reporting scientific results and the need to share best laboratory and clinical practices. In response, more training in rigor and reproducibility, study design, and statistical analysis has been mandated by the National Institutes of Health.^3^ A NINDS task force was one of the first to call on journals to play a more important role in improving the impact of basic and translational research by requiring higher standards of evidence and access to detailed methods, code, and data for publication.^4^ In a climate where increasing research costs and reduced funding have changed the landscape of academic and private sector research, it is even more critical that all research is performed at the highest possible standards. Similarly, drug development costs continue to rise and a high level of scrutiny is given to the design and interpretation of preclinical and clinical studies in the pharmaceutical and biotechnology industries.

The major factors underlying deficiencies in scientific rigor and reproducibility are gaps in training and the absence of widely available best practices. The fields of clinical and translational science are no exception. Clinical and translational research requires an interdisciplinary team of investigators. Clinicians and researchers with training in medicine, pharmacy, pharmacology, pharmaceutical sciences, and bioinformatics bring a diversity of skills and knowledge to the table; this presents both opportunities and challenges. While the breadth of training is critical for the interdisciplinary nature of translational research and medicine, the specialized training in most fields limits the tools and knowledge available to an individual researcher. To address this need, *Clinical and Translational Science* is launching a tutorial section. Focusing on important and timely topics of relevance to the broad field of clinical pharmacology and translational medicine, tutorials will provide practical advice and nuanced perspectives from leaders in the field. The goal is to address the need for best practices, highlight challenges and opportunities for improvement, and to develop a sense of community that is driven by a common goal of improved patient outcomes with more effective and safer medications.

The inaugural tutorial by Arwood et al. provides a detailed framework for implementing pharmacogenetic testing in the clinic. President Obama highlighted the promise of optimized drug therapy in his 2015 State of the Union address and several projects in the Implementing Genomics in Practice (IGNITE) network funded by the National Human Genome Research Institute focus on pharmacogenomic markers. The Pharmacogenomics Research Network funded by the National Institute of General Medical Sciences supported many critical discovery studies that form the basis for several clinically actionable gene–drug associations. After decades of intensive pharmacogenomics discovery research, the time is ripe for translating these findings into the clinic. While widely supported in theory, implementation of pharmacogenetics has been slow. Few clinicians (and even fewer researchers) have the knowledge and training to implement new clinical practices. Implementation to date has been achieved by a lengthy process of trial and error and the development of numerous new tools and standards. Luckily for those who will lead efforts in their own institutions to implement pharmacogenetic testing, the pioneers have carefully documented their path from concept to implementation and generously shared these experiences with the larger community. The pharmacogenetic implementation tutorial in this issue addresses the entire process from selection of gene–drug pairs to outcome analysis in an easy to understand manner.

A regular series of tutorials is planned that will embrace the spirit of education and cooperation that is critical for rigorous scientific research and translation of findings to benefit society. Upcoming topics will consider the use of next generation sequencing in clinical trials, selection of first in human dose, and the development of complementary or companion diagnostics. We welcome your suggestions on topics of interest to clinical and translational scientists.
